# The Causal and Force Perception and Their Perceived Asymmetries in Flight Collisions

**DOI:** 10.3389/fpsyg.2020.01942

**Published:** 2020-08-07

**Authors:** Yuying Wang, Yunyun Chen, Bihua Yan

**Affiliations:** ^1^Shaanxi Key Laboratory of Behavior and Cognitive Neuroscience, School of Psychology, Shaanxi Normal University, Xi’an, China; ^2^Beijing Key Laboratory of Applied Experimental Psychology, National Demonstration Center for Experimental Psychology Education, Faculty of Psychology, Beijing Normal University, Beijing, China

**Keywords:** causal perception, force impression, causal asymmetry, force asymmetry, ecological stimuli

## Abstract

The present study was to investigate causal perception and force perception in ecological objects. Four experiments were designed to compare the perceived causality and force of one of the two objects on the other by changing the property of one or both of the objects involved in the launching effect. Our results support causal asymmetry and force asymmetry, in which the launcher has a greater causal effect and exerts more force on the target. Furthermore, we also found that, the ecological object, which is the airplane in this study, had a greater causal effect and exerted more force, resulting in strengthened asymmetries when the airplane acted as the launcher and weakened asymmetries when the airplane acted as the target. The properties of the object also impacted causal perception by attenuating the effect of the temporal gap on causality. Those results indicate that the airplane is perceived as the main cause for a collision compared with an abstract object. The influence of conceptual knowledge of the object and the sense of agency on changing the degree of perceived causality and force in a particular motion pattern was discussed.

## Introduction

Phenomenal causality, proposed by Michotte (1946/1963), refers to the visual impression that one object causes another to move. Physical interactions that involve non-intentional objects (Scholl and Nakayama, 2002) and stimuli perceived to be animate or intentional objects (Tremoulet and Feldman, 2000) can both bring out causal perception. The launching effect is one among many kinds of phenomenal causalities. In the launching effect, Object A (the launcher) moves toward the stationary Object B (the target). After the contact, the launcher stops moving and the target begins to move along the launcher’s previous direction of movement at the same or a slightly slower speed (Natsoulas, 1961). Observers usually report a causal impression of the target movement caused by the launcher. In addition, they also report a strong force impression that the launcher exerts a certain amount of force on the target in such a collision event.

The velocity ratio may be more important for phenomenal causality and the perceived causality may undergo qualitative change under some conditions. For example, when the target moves at a quicker speed than the launcher, observers usually report another kind of causal perception, the triggering effect; in other words, the target’s motion is initiated or released by the launcher, but its actual motion is perceived as self-generated. However, force impression is determined by both the absolute velocity of the launcher before contact and the absolute velocity of the target after contact (White, 2009). Observers were more likely to report higher force ratings with increases in the velocity of the launcher before contact or the velocity of the target after contact. Force impression may be more sensitive to visual cues and may undergo no qualitative changes. It seems that causal impressions and force impressions may concern different aspects of collision interaction, since many previous studies have found distinct effects of the same variables on causal perception and force impression (e.g., Hubbard and Ruppel, 2013; White, 2014). Despite the differences between the two impressions, causal impression may occur only when observers perceive a certain amount of force, but it is not determined by force impression alone (White, 2014).

Spontaneous reports of observers who view launching effect displays usually mention the launcher’s effect on the target but not the effect of the target on the launcher (e.g., Schlottmann et al., 2006). Ratings of causality and force also show that the launcher is rated as more causal and exerting more force (e.g., White, 2007; Hubbard and Ruppel, 2017) in the typical launching effect. These patterns are referred to as causal asymmetry (White, 2006) and force asymmetry (White, 2012a). In a word, when the roles of cause and effect are assigned to two interacting objects, the importance of the causal object is usually overestimated and that of the effect object was often underestimated in giving rise to the outcome (White, 2006), in spite of the fact that this is not consistent with physical laws.

According to the activity criterion proposed by White (2006), the object that moves first is usually considered to be more active and the causal object, that is, the launcher. White (2012b) called this the “prior motion” hypothesis. However, further studies have shown that this may be limited to the typical launching effect. For example, Hubbard and Ruppel (2013) found that the object that remained intact, or the object that was shattered into fewer fragments, after contact was generally considered to be the causal object and the object was also rated as exerting more force. The shattered object, or the object that was shattered into more fragments, was usually perceived to be the effect object and less forceful, regardless of whether it moved first. Besides, causal asymmetry or force asymmetry may not occur under some conditions. In one study of Hubbard and Ruppel (2017), the launcher stopped moving before it made contact with the target or the target remained stationary after contact. Neither causal asymmetry nor force asymmetry was observed. Hubbard and his colleague suggested that a visible effect of one object on the other may be necessary for the occurrence of the asymmetries. When both objects were in motion before their contact, White (2007) found that the object that moved faster was rated as exerting more force on the other, which showed the force asymmetry. Thus, as White (2006) suggested, the magnitude of the changes in state was also a determining factor for asymmetries (see also Chen and Yan, 2020). Of course, the motion patterns of the two objects in Hubbard’s research were not identical to that in the launching effect. Nonetheless, we focused on the typical launching effect in the present study and investigated how object attributes affect the causal and force asymmetry.

With respect to object attributes, most previous studies have focused on abstract geometric stimuli, which moved in an inanimate way (e.g., Hubbard and Ruppel, 2013; White, 2014) or like an intentional agent (e.g., Falmier and Young, 2008). However, the objects involved in actual collisions are always ecological stimuli, which could be animate objects, such as mice, or inanimate objects, such as airplanes that can be perceived as intentional. In this study, an airplane-shaped object moved in an inanimate way and crashed into, or was crashed into by, another object. Participants were asked to report their visual causal impression and force impression. We assumed that the causal and force perception perceived in a collision involving airplanes may be much stronger, which may influence perceptual asymmetries. On the one hand, evidence from Falmier and Young’s (2008) confirmed the effect of conceptual knowledge about an object on causal perception, in which the researchers found that, when participants were told that the moving object were missiles in their second experiment, the causal ratings were very different from when they were told nothing for the launching effect in their first experiment. Besides, they also found that people more likely accept causality at a distance when the objects moved in an animate manner. However, Falmier and Young did not investigate the effect of conceptual knowledge about an object on causal asymmetry and the airplane is also different from the missile in the sense of agency. On the other hand, Hubbard (2013b) concluded that object properties can influence phenomenal causalities when they are related to the kinematic structures of the stimuli. Properties such as object size (Kotovsky and Baillargeon, 1998) or the direction that the stimuli face (Gao et al., 2010) that are predictive of objects’ motion are more likely to influence phenomenal causalities than, for instance, the color or shape of the stimuli. The airplane has a clear point, and can indicate its own possible direction of movement, which is related to the kinetic structure of the stimulus. With respect to representational momentum (RM), previous studies have found that pointedness affected the magnitude of memory shift (e.g., Nagai and Yagi, 2001) and there are also researches that have reported the relationship between RM and causal perception (e.g., Hubbard et al., 2001; Choi and Scholl, 2006). Thus, airplanes as collision objects may be likely to influence causal impression and force impression and their asymmetries.

## Experiment 1

### Materials and Methods

#### Participants

Fifty-seven undergraduates with normal or corrected-to-normal vision participated for partial credit and were naïve to the purpose. Thirty-two participants (6 males, 26 females) provided judgments of perceived causality, and their mean age was 18.53 years (range = 17–21 years). Twenty-five participants (10 males, 15 females) provided ratings of perceived force, and their mean age was 18.92 years (range = 17–21 years). None of them had ever participated in similar experiments.

#### Apparatus

The stimuli were displayed upon, and data were collected using a Gateway desktop computer connected to a 15-inch color monitor with a refresh rate of 85 Hz and a resolution of 1024 × 768 pixels. Participants’ head and eye movements were not constrained and the viewing distance was about 60 cm. Participants were permitted to adjust this distance slightly for personal comfort.

#### Stimuli: Launcher and Target

The launcher and the target were both black solid disks that were 40 pixels (approximately 9.75 mm) in diameter and were presented against a white background. The target was initially stationary and remained visible within the center of the display prior to contact from the launcher.

We presented the implied motion of the launcher and the target, in which the launcher or the target appeared at one location, vanished, and appeared at another location after 250 ms (cf., Gordon et al., 1990; Hubbard and Ruppel, 2013). There were five successive presentations of the launcher that implied consistent rightwards or leftwards motion before the launcher made contact with the target. These successive presentations were referred to as launcher-inducing stimuli. At the moment of contact, the launcher immediately stopped moving, while the delay in the movement of the target in a similar direction was 30 or 150 ms. There were four successive presentations of the target, which were referred to as target-inducing stimuli. For rightwards (or leftwards) motion, the first launcher-inducing stimulus appeared 272 pixels (approximately 66.25 mm) away from the left (or the right) side of the display. The vertical coordinates of the inducing stimuli were approximately centered on the vertical axis of the display. In addition, the horizontal coordinates of each successive inducing stimulus were located 50 pixels (approximately 12.19 mm) to the center of the previous inducing stimulus. After contact from the launcher, the target began to move. The horizontal coordinates of each successive target-inducing stimulus were also located 50 pixels to the center of the previous target-inducing stimulus. The inter-stimulus interval (ISI) between inducing stimuli was always 250 ms. However, the time of each presented inducing stimulus was not always the same. We designed three different speed combinations by varying presenting time of the two kinds of inducing stimuli. For the fast/slow speed combination, each launcher-inducing stimulus was presented for 150 ms, while each target-inducing stimulus was presented for 300 ms. For the fast/fast speed condition, both kinds of inducing stimuli were presented for 150 ms, and for the slow/slow speed conditions, both kinds of inducing stimuli were presented for 300 ms.

#### Rating Scale

The causality rating scales were adapted from those used in Zhou et al. (2012), and the force rating scales were adapted from those used in previous studies (White, 2007, 2009; Hubbard and Ruppel, 2013, 2017). Both kinds of scales included two questions. Participants who gave ratings of causality were asked: “Did the first (or the second) object cause any motion or change in the second (or the first) object?” They were instructed to rate 1 for YES or 0 for NO. Participants who gave ratings of force were asked: “How much force did the first object (or the second object) exert on the second object (or the first object)?” They were instructed to rate on a 0 to 100 scale; the more force they perceived one object exerting on the other, the higher the number they should indicate up a maximum of 100. We have decided to use a categorical measure for causal impressions and a continuous rating for force impressions, because the YES/NO judgment could help us understand the probability of one condition being perceived as a causal event; that is, whether perceived causality exists or not. With regard to force perception, we want to figure out the magnitude of it.

#### Procedure

**FIGURE 1:**
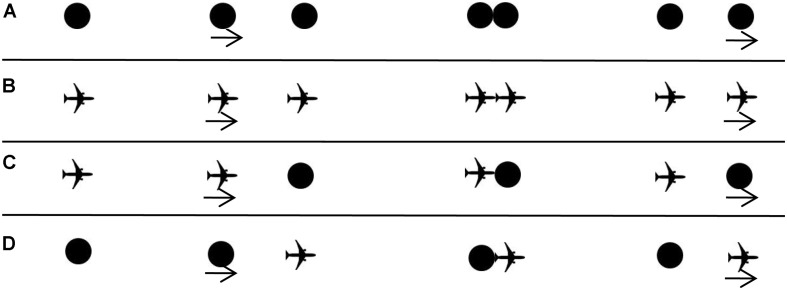
First, the target appears, followed by the launcher’s entry from left (or right) approaching the target. At the moment of contact, the launcher immediately stops moving and the target begins to move a moment later in the same direction. **(A)** Procedure of Experiment 1. **(B)** Procedure of Experiment 2. **(C)** Procedure of Experiment 3. **(D)** Procedure of Experiment 4.

As shown in Figure 1A, there was a 1000 ms delay after participants pushed a designated key to begin the trial, after which the target appeared. For the fast/fast and fast/slow speed combinations, the launcher appeared and moved toward the target after 150 ms, while for the slow/slow speed combination, the launcher approached the target after 300 ms. The launcher and target were visible for 1000 ms after the target motion ceased, after which the launcher and target simultaneously disappeared. Following this, the rating scale appeared near the center of the display and remained visible until participants entered their rating into a keyboard attached to the desktop computer. Participants then initiated the next trial.

#### Design

One group of participants rated causality and another rated force. Each participant completed two blocks of trials. In one block, participants rated the causality or force of the first object (the launcher) on the second object (the target). In another block, participants rated the causality or force of the target on the launcher. The order of the two blocks was random. Each participant received 48 trials [3 speed combinations (fast/fast; fast/slow; slow/slow) × 2 directions (leftwards, rightwards) × 2 delays (30, 150 ms) × 4 replications] in each block.

Participants began with a practice session comprising 12 trials that included examples of each experimental condition.

### Results

**FIGURE 2:**
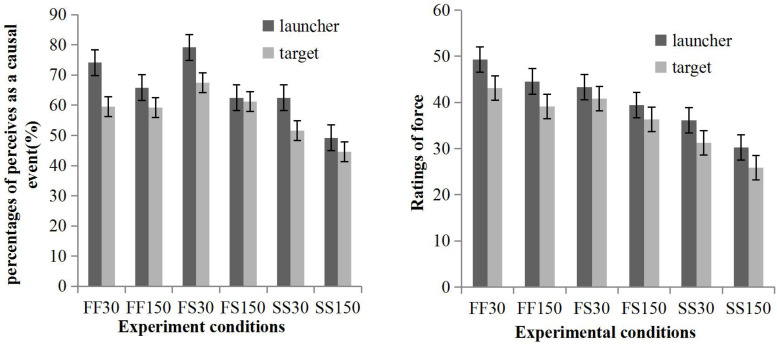
The percentage of trials perceived as causal events **(left panel)** and the ratings of force **(right panel)** varied in different experimental conditions. “FF” indicates when the launcher and the target both moved fast, in which each inducing stimulus was presented for 150 ms. “FS” indicates when the launcher moved fast while the target moved slowly, in which each launcher-inducing stimulus was presented for 150 ms, while each launcher-inducing stimulus was presented for 300 ms. “SS” indicates that the launcher and the target both moved slowly, in which each inducing stimulus was presented for 300 ms. The number “30” indicates that the target began to move after 30 ms after the launcher made contact with the target and “150” indicates that the target began to move 150 ms later after contact. Error bars represent standard error. Directions were merged because no main effect was found.

The percentages of trials that were perceived as causal events and ratings of force are shown in Figure 2 and were analyzed in separate repeated measures ANOVAs: 2 (source: launcher, target) × 3 (speed combination: fast/fast, fast/slow, slow/slow) × 2 (direction: rightwards, leftwards) × 2 (delay: 30, 150 ms).

#### Percentages of Trials Perceived as a Causal Event

The main effect of the source was significant, *F*(1, 31) = 4.57, *p* = 0.041, *MSE* = 2346.23, with more trials perceived as causal events when the causality of the launcher on the target was indicated (*M* = 66.47%, *SE* = 3.96), than when causality of the target on the launcher was indicated (*M* = 59.00%, *SE* = 4.19). Speed combination was significant, *F*(1.51, 46.94) = 15.18, *p* < 0.001, *MSE* = 1185.20, and pairwise comparisons with Bonferroni correction showed that the percentage of trials perceived as causal events in the fast/fast condition (*M* = 65.94%, *SE* = 3.94) was higher than that in the slow/slow condition (*M* = 53.22%, *SE* = 4.19). The percentage of trials perceived as causal events in the fast/slow condition was also higher than that in the slow/slow condition (*M* = 69.04%, *SE* = 4.11). Delay was significant, *F*(1, 31) = 6.65, *p* = 0.015, *MSE* = 2291.65; a 30 ms delay (*M* = 67.19%, *SE* = 3.79) resulted in a higher percentage of trials perceived as causal events than a 150 ms delay (*M* = 58.28%, *SE* = 4.33). No other main effects or interactions approached significance (*p* > 0.05).

#### Ratings of Force

The main effect of source was significant, *F*(1, 24) = 4.53, *p* = 0.044, *MSE* = 644.36, with the force exerted by the launcher (*M* = 40.50, *SE* = 3.53) rated as higher than the force exerted by the target (*M* = 36.09, *SE* = 3.70). Speed combination was significant, *F*(1.48, 35.63) = 21.54, *p* < 0.001, *MSE* = 567.75, and pairwise comparisons with Bonferroni correction revealed that all pairwise comparisons between fast/fast condition (*M* = 44.03, *SE* = 22.36), fast/slow condition (*M* = 39.98, *SE* = 20.62), and slow/slow condition (*M* = 30.88, *SE* = 20.03) were significant. Delay was significant, *F*(1, 24) = 6.45, *p* = 0.018, *MSE* = 523.55; a 30 ms delay (*M* = 40.67, *SE* = 3.67) resulted in higher ratings than a 150 ms delay (*M* = 35.92, *SE* = 3.50). No other main effects or interactions approached significance (*p* > 0.05).

### Discussion

#### Perceived Causality

In a replication of previous research (White, 2006, 2007), the launcher was more likely to be perceived as making the target move, while the target was less likely to be perceived as causing the launcher’s stop of movement. This is consistent with the causal asymmetry hypothesis.

Both velocity and temporal contiguity have a strong impact on causal perception. More trials in the fast/fast and fast/slow speed combinations were perceived as causal events than in the slow/slow condition, while the percentages of trials perceived as causal events in these two conditions were not significantly different. Our study focused on whether a causal launching event occurs, and the results reveal that the launching effect is more likely to occur as the speed of the launcher, prior to contact, increases (Michotte, 1946/1963; Natsoulas, 1961). Natsoulas (1961) found that speed ratio was more important in determining the quality of the phenomenal causality. Our observation further indicates that the speed of the launcher is more critical in determining the magnitude of the launcher effect (see also White, 2014). Increases in the temporal gap between when the launcher stopped moving and when the target began moving decrease the percentage of trials perceived as causal events. About 60% of the trials (58.28%) in the 150 ms delay condition was perceived as a causal launching. This seems to be in contrast with previous findings (cf., Michotte, 1946/1963, White, 2014), in which reporting of the launching effect dropped rapidly and reached 0% of trials with a delay of 126 ms. However, other authors have also reported the occurrence of causal impressions with delays longer than 200 ms, depending on the presentation conditions (Schlottmann and Shanks, 1992; Guski and Troje, 2003; Schlottmann et al., 2006). Therefore, it is possible that causal impression could occur at an even longer time delay.

#### Perceived Force

White (2009) established that, in the launching effect, the force exerted by the launcher on the target was higher than the force exerted by the target on the launcher, showing a significant perceived force asymmetry. Our result is consistent with this finding. Besides, speed combination significantly affects the perceived force. When the launcher moved faster before contact or the target moved faster after contact, the force perceived was stronger, indicating that the force impression tends to be strengthened as the absolute velocity of the launcher before contact and that of the target after contact increase. This is consistent with previous findings (White, 2007, 2009). However, the effects of object velocity on causal and force impressions were not exactly the same and we will provide a possible explanation for this in the general discussion. We found that time contiguity affects force perception in a way similar to the experiments in previous research (White, 2014). When the delay between the moment at which the launcher contacts the target and the moment at which the target begins to move was longer, the ratings of force declined. The mean rating of force at a longer delay was 35.92. It seems that there is a weak force impression at longer time delays. Nonetheless, the results of Experiment 1 reveal that the impression in the launching effect shows perceptual asymmetry, with the launcher, who moves first, perceived as more causal and exerting more force.

## Experiment 2

### Materials and Methods

#### Participants

The participants in Experiment 2 were 48 undergraduates with normal or corrected-to-normal vision, participating for partial credit. Twenty-seven participants (4 males, 23 females) provided judgments of causality, and their mean age was 18.33 years (range = 17–21 years). Twenty-one participants (5 males, 16 females) gave ratings of force, and their mean age was 18.62 years (range = 17–22 years). None of them had participated in similar experiments.

#### Apparatus, Stimuli, Procedure, and Design

The launcher and the target were both black, solid airplane shapes and were 50 pixels (approximately 12.19 mm) in height and width as shown in Figure 1B. For rightwards (or leftwards) motion, the first launcher-inducing stimulus was located 262 pixels (approximately 63.75 mm) away from the left (or right) side of the display. Other experimental details were as for Experiment 1.

### Results

**FIGURE 3:**
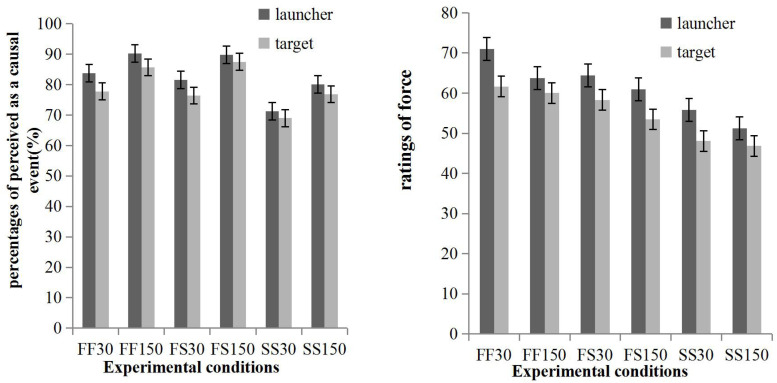
The percentage of trails perceived as causal events **(left panel)** and the ratings of force **(right panel)** in the interaction of two airplanes.

The percentages of trials perceived as causal events and ratings of force are shown in Figure 3 and were analyzed in separate repeated measures ANOVAs: 2 (source: launcher, target) × 3 (speed combination: fast/fast, fast/slow, slow/slow) × 2 (direction: rightwards, leftwards) × 2 (delay: 30, 150 ms).

#### Percentages of Trials Perceived as a Causal Event

The main effect of the source was significant, *F*(1, 26) = 6.35, *p* = 0.018, *MSE* = 395.30, with more trials perceived as causal events when the causality of the launcher on the target was rated (*M* = 82.79%, *SE* = 3.00) than when the causality of the target on the launcher was rated (*M* = 78.86%, *SE* = 3.06). Speed combination was significant, *F*(1.28, 33.24) = 8.18, *p* = 0.001, *MSE* = 844.20, and pairwise comparisons with Bonferroni correction showed that the percentage in the fast/fast condition (*M* = 84.38%, *SE* = 2.95) was higher than that in the slow/slow condition (*M* = 74.31%, *SD* = 4.04), and that the percentage in the fast/slow (*M* = 83.80%, *SE* = 2.93) condition was higher than that in the slow/slow condition. No other main effects or interactions approached significance (*p* > 0.05).

#### Ratings of Force

The main effect of source was significant, *F*(1, 20) = 16.74, *p* = 0.001, *MSE* = 313.94, with the force exerted by the launcher (*M* = 61.19, *SE* = 2.65) rated as higher than the force exerted by the target (*M* = 54.73, *SE* = 2.52). Speed combination was also significant, *F*(1.52, 30.45) = 28.37, *p* < 0.001, *MSE* = 372.03, and pairwise comparisons with Bonferroni correction showed that all pairwise comparisons between fast/fast (*M* = 64.13, *SE* = 2.23), fast/slow (*M* = 59.27, *SE* = 2.52), and slow/slow conditions (*M* = 50.48, *SE* = 3.20) were all significant. In addition, delay was significant, *F*(1, 20) = 10.24, *p* = 0.004, *MSE* = 180.99, with force rated in the 30 ms delay (*M* = 59.88, *SE* = 2.47) as higher than in the 150 ms delay (*M* = 56.04, *SE* = 2.59). No other main effects or interactions approached significance (*p* > 0.05).

### Discussion

The two airplane-shaped objects in Experiment 2 produced results similar to those produced by the two disks in Experiment 1, except that temporal contiguity in Experiment 2 did not affect causal perception. Observers provided similar responses when the target began to move after 30 or 150 ms. One possible explanation could be that an ecological object may attenuate the impact of the temporal delay on the causal impression. In the collision of physical world, it is very common to observe the effect object moving after some time, depending on the mass ratio or some other factors, thus resulting in a relatively higher likelihood of reporting a perceived launching effect. Velocity has a consistent impact on causal perception and force perception, respectively. What is important is that the causal asymmetry and force asymmetry are profound, indicating that, in a relatively real collision, we also tend to perceive the launcher as the causal object and as exerting more force.

## Comparison Between Data of Experiment 1 and Experiment 2

The only difference between Experiments 1 and 2 is the difference in stimulus materials. This was helpful to establish the effect of object property on the perception of causality and the force perception to combine the two sets of data.

The percentage of trials perceived as causal events and ratings of force were analyzed in separate group (Experiments 1, 2) × source × speed combination × delay repeated measures ANOVAs.

### Percentages of Trials Perceived as a Causal Event

The main effect of the group was significant, *F*(1, 57) = 14.02, *p* < 0.001, *MSE* = 728.17, with more trials perceived as causal events in Experiment 2 (*M* = 80.83%, *SE* = 3.56) than in Experiment 1 (*M* = 62.73%, *SE* = 3.27), indicating that the object property of the airplane shape impacts causal perception by increasing the likelihood of reporting causal launching in the interaction between two airplanes. The interaction of delay × group was significant, *F*(1, 57) = 7.62, *p* = 0.008, *MSE* = 1728.56, and simple effect analysis showed that, when the target started to move after 30 ms, the percentages in Experiments 1, 2 were not different. However, when the target started to move after 150 ms, more trials in Experiment 2 were perceived as causal events (*M* = 85.03%, *SE* = 3.98). This confirms that the shape of the airplane attenuates the effect of delay on causal perception. Even when the target airplane started to move after a longer period of time, it was still easily perceived as a causal event. What is important was that the interaction of source and group was not significant, indicating that the causal asymmetry in Experiments 1, 2 was not different, *F*(1, 57) = 0.22, *p* > 0.6.

### Ratings of Force

The main effect of the group was significant, *F*(1, 44) = 19.94, *p* < 0.001, *MSE* = 221.43; force rated in Experiment 2 (*M* = 57.96, *SE* = 3.25) was higher than that in Experiment 1 (*M* = 38.29, *SE* = 2.98), showing that the airplane-shaped object also strengthens the force perception. It was important that source × group interaction was not significant, *F*(1, 43) = 0.58, *p* > 0.45; that is, the magnitude of perceived force asymmetry was similar no matter whether the launcher and the target were both abstract stimuli or airplanes.

## Experiment 3

### Materials and Methods

#### Participants

Participants were 69 undergraduates with normal or corrected-to-normal vision, participating for partial credit. Forty participants (8 males, 32 females) provided judgments of causality, and their mean age was 18.68 years (range = 17–21 years); 29 participants (10 males, 19 females) provided ratings of force, and their mean age was 18.76 years (range = 18–21 years). They were all right-handed and none of them had participated in similar experiments before.

#### Apparatus, Stimulus Materials, Procedure, and Design

The launcher in Experiment 3, as shown in Figure 1C, was the same airplane shape adopted in Experiment 2. For rightwards (or leftwards) motion, the first launcher-inducing stimulus was located 267 pixels (approximately 65 mm) away from the left (or right) side of the display. Other experimental details were as for Experiment 1.

### Results

**FIGURE 4:**
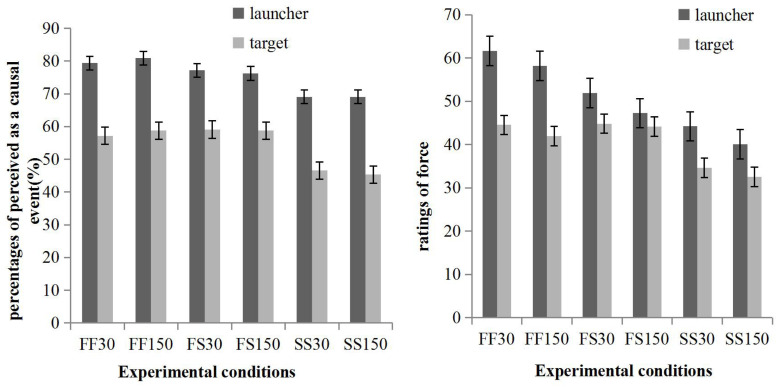
The percentage of trails perceived as causal events **(left panel)** and the ratings of force **(right panel)** perceived in Experiment 3.

The percentages of trials perceived as causal events and ratings of force are shown in Figure 4 and were analyzed in separate repeated measures ANOVAs: 2 (source: launcher, target) × 3 (speed combination: fast/fast, fast/slow, slow/slow) × 2 (direction: rightwards, leftwards) × 2 (delay: 30, 150 ms).

#### Percentages of Trials Perceived as a Causal Event

The main effect of the source was significant, *F*(1, 39) = 29.83, *p* < 0.001, *MSE* = 3562.77; more trials were perceived as causal events when participants rated the causality of the launcher on the target (*M* = 75.31%, *SE* = 2.37) than when they rated the causality of the target on the launcher (*M* = 54.27%, *SE* = 4.05). The speed combination was significant, *F*(1.73, 67.64) = 13.39, *p* < 0.001, *MSE* = 962.34, and pairwise comparisons with Bonferroni correction showed that the percentages of trials perceived as causal events in the fast/fast (*M* = 69.06%, *SE* = 2.82) and slow/slow (*M* = 57.50%, *SE* = 3.20) conditions were significantly different. The case was similar between the fast/slow (*M* = 67.81%, *SE* = 3.11) and slow/slow conditions. No other main effects and two-dimensional interactions approached significance (*p* > 0.05).

#### Ratings of Force

The main effect of source was significant, *F*(1, 28) = 31.43, *p* < 0.001, *MSE* = 565.59, with the force exerted by the airplane (*M* = 50.55, *SE* = 2.31) rated as higher than the disk (*M* = 40.44, *SE* = 1.94). Speed combination was significant, *F*(2, 56) = 35.23, *p* < 0.001, *MSE* = 321.36, and pairwise comparisons with Bonferroni correction showed that all pairwise comparisons between fast/fast condition (*M* = 51.58, *SE* = 2.43), fast/slow condition (*M* = 47.03, *SE* = 2.19), and slow/slow condition (*M* = 37.87, *SE* = 1.81) were significant. Delay was significant, *F*(1, 28) = 5.41, *p* = 0.028, *MSE* = 274.66; force perceived in the 30 ms delay (*M* = 46.95, *SE* = 2.08) was greater than in the 150 ms delay (*M* = 44.03, *SE* = 1.99). The interaction of source and speed combination was significant, *F*(2, 56) = 5.73, *p* = 0.005, *MSE* = 357.51, and simple effects analysis revealed that the force exerted by the launcher rated in the fast/fast and slow/slow speed combinations was higher than the force exerted by the target (*p* < 0.001, respectively). However, the force exerted by the launcher and the target rated in the fast/slow condition was only marginally different, *p* = 0.054. No other main effect or interaction approached significance (*p* > 0.05).

### Discussion

In Experiment 3, we replaced the launcher disk with a stimulus shaped like an airplane and found that the launcher airplane was perceived as more causal than the target and exerted more force on the target, which showed substantial causal asymmetry and force asymmetry in general. Object velocity had a consistent, albeit slightly different, impact on causal and force perception as shown in Experiments 1 and 2. However, temporal contiguity had a consistent impact on force impression while not on causal perception. In interactions involving the airplane-shaped stimuli, causal perception was not different between the 30 and 150 ms delay conditions, which suggests the attenuating effect of the delay on causal impression in interaction with an airplane.

## Comparison of Data Between Experiment 1 and Experiment 3

Percentages of trials perceived as causal events and ratings of force were analyzed in separate repeated measures ANOVAs: group (Experiments 1, 3) × source × speed combination × delay. Similarly, we were concerned with group effect and two-dimensional interaction effect related to group.

### Percentages of Trials Perceived as a Causal Event

The interaction of source × group was significant, *F*(1, 70) = 6.49, *p* = 0.013, *MSE* = 1512.08, and simple effects analysis revealed that more trials in Experiment 3 were perceived as causal events compared with those in Experiment 1 when participants rated the causality of the launcher on the target, *p* = 0.049. In addition, when participants were asked to rate the causality of the target on the launcher, the two groups of participants gave similar judgments. The results indicate that causal asymmetry was greater in Experiment 3, resulting from a strong causal effect of the airplane acting as the launcher.

### Ratings of Force

The interaction of source × group was significant, *F*(1, 52) = 4.34, *p* = 0.042, *MSE* = 300.97, and simple effects analysis revealed that the force exerted by the launcher rated in Experiment 3 was greater than that in Experiment 1, *p* = 0.018. In addition, the force exerted by the target rated in Experiments 1, 3 was not different. The results revealed that the magnitude of the force asymmetry in Experiment 3 was also much greater than that in Experiment 1. We found that the airplane shape strengthened the force impression in Experiment 2 and this was consistent in Experiment 3 when the launcher was airplane-shaped. The force asymmetry was strengthened because, when the airplane acted as the launcher, observers perceived much greater force.

## Experiment 4

### Materials and Methods

#### Participants

There were 49 participants with normal or corrected-to-normal vision, participating voluntarily and naïve to the purpose of the experiment. Twenty of them (6 males, 14 females) provided judgment of causality, and their mean age was 18.85 years (range = 17–23 years). Twenty-nine of them provided ratings of force (8 males, 21 females), and their mean age was 19.07 years (range = 17–23 years). They were all right-handed and had never participated in similar experiments.

#### Apparatus, Stimulus Materials, Procedure, and Design

The target was the airplane with a height and a width of 50 pixels as shown in Figure 1D. For rightwards (or leftwards) motion, the first launcher-inducing stimulus was located 267 pixels (approximately 65 mm) away from the left (or right) side of the display. Other experimental details were as for Experiment 1.

### Results

**FIGURE 5:**
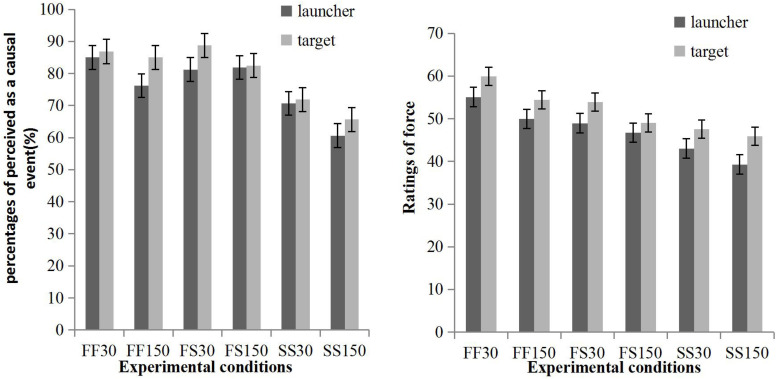
Percentage of trails perceived as causal events **(left panel)** and ratings of force **(right panel)** as a function of different conditions in Experiment 4.

The percentages of trials perceived as causal events and ratings of force are shown in Figure 5 and were analyzed in separate repeated measures ANOVAs: 2 (source: launcher, target) × 2 (speed combination: fast/fast, fast/slow, slow/slow) × 2 (direction: rightwards, leftwards) × 2 (delay: 30, 150 ms).

#### Percentages of Trials Perceived as a Causal Event

The main effect of speed combination was significant, *F*(1.30, 24.71) = 10.56, *p* < 0.001, *MSE* = 2050.79, and pairwise comparisons with Bonferroni correction showed that more trials in the fast/fast (*M* = 83.28%, *SE* = 2.88) and fast/slow (*M* = 83.59%, *SE* = 2.48) speed combination were perceived as causal events than in the slow/slow (*M* = 67.19%, *SE* = 5.21) speed combination. No other main effects or interactions approached significance (*p* > 0.05).

#### Ratings of Force

Speed combination was significant, *F*(2, 56) = 16.59, *p* < 0.001, *MSE* = 415.41, and least-square comparison revealed that all pairwise comparisons between fast/fast (*M* = 54.85, *SE* = 3.54), fast/slow (*M* = 49.65, *SE* = 2.57), and slow/slow (*M* = 43.96, *SE* = 2.98) speed combination conditions were significant. Delay was also significant, *F*(1, 28) = 7.66, *p* = 0.010, *MSE* = 340.81, with force rated in the 30 ms delay (*M* = 51.42, *SE* = 2.96) higher than force rated in 150 ms (*M* = 47.55, *SE* = 3.16). No other main effects or interaction were significant.

### Discussion

Object velocity had a similar influence on the causal impression and the likelihood of reporting a launching effect increased with increases in the speed of the launcher before contact. Delay did not affect causal perception when one of the objects was shaped like an airplane. The effects of object velocity and temporal contiguity on force ratings remained consistent as in the previous three experiments, with the faster the movement of the object and the shorter the time before the target began to move, the stronger the perceived force impression.

The very important, but not surprising, finding of Experiment 4 was that the main effect of the source was not significant for causal judgments and force ratings when the airplane-shaped object acted as the target. There are two possible reasons for this. One possible explanation is that an airplane’s motion can be self-propelled, which may result in a relatively lower causal and force perception when the target was the airplane. If this is correct, the causality of the launcher on the target should be weakened. Another reason may be that the airplane shape itself is perceived as more dominant, as in Experiments 2, 4, and participants are more likely to notice the effect of the airplane even when it acts as an inactive target, thus resulting in weaker perceptual asymmetry. If this is correct, the causality of the target airplane on the launcher should be far stronger compared with previous experiments.

## Comparison of Data Between Experiment 1 and Experiment 4

The percentage of trials perceived as a causal event and rating of force were analyzed in separate repeated measures ANOVAs: group (Experiments 1, 4) × source × speed combination × delay. Group effect and two-dimensional interaction effect related to group were concerned.

### Percentages of Trials Perceived as a Causal Event

The main effect of the group was significant, *F*(1,50) = 8.65, *p* = 0.005, *MSE* = 332.49; more trials were perceived as a causal event in Experiment 4 (*M* = 78.02%, *SE* = 4.08) compared with that in Experiment 1 (*M* = 62.73%, *SE* = 3.22). The interaction of source × group was significant, *F*(1, 50) = 5.08, *p* = 0.029, *MSE* = 985.16, and a simple effects analysis revealed that, when participants were asked to judge the causality of the target on the launcher, more trials were perceived as causal events in Experiment 4 compared to that in Experiment 1, *p* = 0.001. In addition, when participants were asked to judge the causality of the launcher on the target, the percentages of trial perceived as causal events were not different between the two experiments. Nonetheless, the significant interaction of source and group showed that more trials in Experiment 4 were perceived as causal events when participants were asked to judge the causality of the target on the launcher.

### Ratings of Force

The main effect of the group was significant, *F*(1, 52) = 6.07, *p* = 0.017, *MSE* = 277.04, with force rated in Experiment 4 (*M* = 49.49, *SE* = 3.09) higher than that in Experiment 1 (*M* = 38.29, *SE* = 3.33). The interaction of source and group was significant, *F*(1, 52) = 7.31, *p* = 0.009, *MSE* = 448.12, and a simple effects analysis revealed that the rating of the force exerted by the launcher was not different between Experiment 1 and 4, while the rating of the force exerted by the target in Experiment 4 was higher than that in Experiment 1, *p* = 0.003, showing that the target airplane was perceived as more forceful than the target disk.

## General Discussion

### Variables That Influence Causal and Force Perception in Flight Collision

#### Speed

With respect to causal impression, the four experiments showed that all comparisons between fast/fast, fast/slow, and slow/slow speed conditions were significantly different, with the exception of the fast/fast and fast/slow conditions. In the present study, when the launcher moved faster before it made contact with the target, the likelihood of the participant reporting a launching effect was higher. It appears that the velocity of the launcher before contact is more critical for the occurrence of the launching effect. In terms of perceived force, the faster the launcher moved prior to contact and the faster the target moved after contact, the greater the force is perceived, indicating that perceived force is more dependent on both of the two objects’ absolute velocities. When we replaced the launcher or the target with the airplane-shaped object, the effect of speed on the perception of causality and force perception remained stable. However, velocity has different effects on causal perception and force perception, respectively. As White (2014) has argued, causality may emphasize the relation between input and outcome in which something happens to the target when the launcher acts thereon in some way. Thus, in terms of causality, the point is whether there is perceptible change, such as motion or deformation, not how much change is perceived in the object acted upon. Since the initially moving launcher stopped moving and the initially stationary target started to move eventually, observers may focus on the launcher. When the launcher moves faster, it may be considered to change the motion of the target more likely and the causal relation may be much easier to be perceived. On the other hand, when the launcher with a faster speed stops moving, it may be more likely to be perceived as being stopped by the target. However, the emphasis of force impression may be the input side (White, 2014). When asked how much force the launcher or the target has on each other, it is very likely for observers to take how much change is perceived in both of the two interacting objects into consideration; thus, both velocities of the two objects are important. Nonetheless, the findings reflect disassociation of perceived causality and perceived force, which is consistent with White’s findings (White, 2011b, 2014).

#### Temporal Contiguity

In a replication of previous studies (e.g., White, 2014), in the interaction of two disks, participants are less likely to report a launching effect and perceive less force when the latency between when the launcher stops moving and when the target begins moving increases. For interactions involving airplane-shaped objects (Experiments 2–4), it appears that object properties of the two moving objects do affect the perceived causal relationship between them, by attenuating the impact of the temporal gap. However, the perceived force was still lower with longer temporal delays. One explanation may be that an airplane as a machine operated by human pilots may elicit the sense of intentionality or agency, which attenuates the impact of the temporal delay. Therefore, our results may reveal that people more readily accepted causality with a temporal delay when the objects are airplanes, which may be intentional. Another explanation may be that it is more possible to take resistance, such as air resistance, into consideration with respect to the physical interaction of relatively real objects. According to the naïve impetus theory (Mccloskey, 1983), for a stationary target to move, the impetus exerted thereon should be greater than the resistance exerted thereon to prevent it from moving. When the impetus decreases below the resistance, the motion of the object ceases. Some researchers have discussed that launching effect could reflect an attribution of naïve impetus imparted from the launcher to the target and that impetus then dissipated with subsequent target motion (Hubbard, 2013a). However, the impetus could dissipate immediately when imparted to the target. Therefore, when the latency before the object begins to move increases, the impetus may dissipate already, which results in a lower level of force perception. However, the object still began to move eventually, indicating that the impetus was still greater than the resistance. Thus, individuals were still more likely to perceive it as a causal event.

#### Object Property

Evidence shows that object property influences causal impression and force impression. Firstly, more trials in Experiment 2 were perceived as causal events. Secondly, when the airplane-shaped object acted as the launcher in Experiment 3, the causality of the launcher on the target was far stronger than when the launcher was disk-shaped, as in Experiment 1. Thirdly, when the airplane-shaped object acted as the target in Experiment 4, the causal effect of the target on the launcher was stronger than when the target was disk-shaped, as in Experiment 1. These results suggest that participants are more likely to notice the role of the ecological object in a collision. Michotte (1946/1963) argued that phenomenal causality was based on the kinetic structure, such as its absolute size or facing direction, of the stimulus. The structural properties of an airplane usually indicate its possible moving direction. When an airplane-shaped object acts as the launcher or the target, it is more likely to be predictive of its own moving direction and, thus, more likely to influence phenomenal causality. The airplane shape also enhances force perception, which is manifest especially in the fact that the perceived force by the participants in Experiment 2 was stronger than that in Experiment 1. In Experiment 3, when the launcher was airplane-shaped, the force exerted by the launcher on the target was far greater, while in Experiment 4, when the target was airplane-shaped, the force exerted by the target on the launcher was far greater compared to that in Experiment 1. Even in some cases, the results showed a reversed force asymmetry in which the force exerted by the target on the launcher was more than the force exerted by the launcher on the target. These results confirm that the conceptual knowledge of an object affects force perception. One explanation may be that, compared with the disk-shaped object, the airplane shape is more likely to move and the interaction involving airplanes will produce a more vivid collision experience. Therefore, the airplane-shaped object is more likely to exert force on the other object even when acting as the inactive target. If White’s argument that our understanding of causality comes from the experience of force acting on objects (White, 2009, 2012a) is correct, then this may partly account for the strengthened causal perception in interactions involving airplane-shaped objects. Another explanation may be that the different shapes of the stimuli induce, for example, different assumptions about the mass of the depicted objects based on their experiences in the physical world. Michele (2018) found that the implied mass of the colliding objects would affect causal perception. Thus, in this study, heavier airplanes could make lighter disks move or stop moving easily and exert more force on the disk. On the other hand, when heavier objects move after collision, heavier objects would be perceived as being exerted more force and caused by collision more likely. Finally, airplanes are operated by the pilots; thus, these results also suggest that people could interpret the airplane-shaped object as an agent that has self-propelled and intentional motion. The motion of the airplane may reflect the intention of the pilots. Therefore, these results may elucidate that causal perception and force perception are cognitively penetrated.

#### Direction

On the whole, the manipulation of the direction of movement did not affect the causal impression and force impression. We presented both left-to-right and right-to-left motion, and the percentage of reporting a causal launching effect and force ratings for rightwards motion were not different from those for leftwards motion. Although Hubbard and Ruppel (2017) found that perceived causality and force were stronger for rightwards motion, the difference was not very significant. Two differences may account for this contrast in our studies. The target in Hubbard’s experiment remained stationary throughout and the launcher stopped moving at a distance from the target or at contact with the target. In another study (Hubbard and Ruppel, 2018), the effects of direction on perceived causality and force were not as consistent. Thus, with the available evidence, it is difficult to know conclusively how the direction of movement impacts causal impression and force impression. Nonetheless, the effect of direction on causal perception and force perception may be dependent on different contexts. In a typical launching effect, the direction is less likely to influence causal perception and force perception.

### Perceived Causal and Force Asymmetry in Flight Collision

Previous studies have found causal and force asymmetry in various phenomenal causalities such as in launching effect and shattering effect (e.g., White, 2011a; Hubbard and Ruppel, 2013). White (2012b) believed that, in the typical launching effect, the launcher was generally considered to be the causal object, have a greater causal effect, and exert more force. However, our study found that causal and force asymmetry was influenced by object property in the typical launching effect. Although the magnitudes and directions of the causal and force asymmetry in Experiment 2 were not different from those in Experiment 1, the causality and force perceived were greater. In Experiment 3, the airplane-shaped object, acting as the launcher, enhanced the causal effect and force effect of the launcher, thereby strengthening the causal and force asymmetry. At the same time, the airplane acting as the target enhanced the causal perception and force perception of the target in Experiment 4, thereby weakening the causal and force asymmetry. It appears that perceptual asymmetries are influenced by the airplane-shaped object in a similar way; that is, the airplane shape is more likely to play a causal role in a collision and exerts much more force. The airplane-shaped object may be more closely related to physical collisions since an airplane can usually move along the direction indicated by the airplane’s nose. Besides, an airplane can move intentionally because it is operated by pilots. Therefore, when the airplane-shaped object acts as the launcher, it is more likely to cause the motion of the target intentionally, and when the airplane acts as the inactive target, it is more likely to be considered to stop the launcher’s motion.

## Conclusion

In conclusion, object velocity has a strong, consistent but slightly different effect on causal and force impression. In addition, the speed of the launcher before contact is more important for causal impression, while, for force impression, the velocities of both interacting objects are equally important. The effect of temporal contiguity on causal impression is influenced by object property in the way of attenuating the impact of the temporal gap. Object property that is related to kinetic structure or intentionality can also influence the causal and force perception per se and therefore influence the perceived causal asymmetry and force asymmetry.

## Data Availability Statement

The datasets generated for this study are available at: https://github.com/dao2shui/CFPFC.

## Ethics Statement

Ethical review and approval was not required for the study on human participants in accordance with the local legislation and institutional requirements. The patients/participants provided their written informed consent to participate in this study.

## Author Contributions

YW and YC organized the database and performed the statistical analysis. YC wrote the first draft of the manuscript. YW wrote sections of the manuscript. All authors contributed to the conception and design of the study, manuscript revision, read and approved the submitted version.

## Conflict of Interest

The authors declare that the research was conducted in the absence of any commercial or financial relationships that could be construed as a potential conflict of interest.
